# Facial functional networks during resting state revealed by thermal infrared imaging

**DOI:** 10.1007/s13246-023-01321-9

**Published:** 2023-08-29

**Authors:** Daniela Cardone, Francesco Cerritelli, Piero Chiacchiaretta, David Perpetuini, Arcangelo Merla

**Affiliations:** 1 Department of Engineering and Geology, University G. d’Annunzio of Chieti-Pescara, 65127 Pescara, Italy; 2 Clinical Human-Based Department, Foundation COME Collaboarion, 65121 Pescara, Italy; 3 Department of Innovative Technologies in Medicine and Odontoiatry, University G. d’Annunzio of Chieti-Pescara, Chieti, Italy

**Keywords:** Seed correlation map, Thermal infrared imaging, Facial physiology, Galvanic skin response, Image warping

## Abstract

In recent decades, an increasing number of studies on psychophysiology and, in general, on clinical medicine has employed the technique of facial thermal infrared imaging (IRI), which allows to obtain information about the emotional and physical states of the subjects in a completely non-invasive and contactless fashion. Several regions of interest (ROIs) have been reported in literature as salient areas for the psychophysiological characterization of a subject (i.e. nose tip and glabella ROIs). There is however a lack of studies focusing on the functional correlation among these ROIs and about the physiological basis of the relation existing between thermal IRI and vital signals, such as the electrodermal activity, i.e. the galvanic skin response (GSR). The present study offers a new methodology able to assess the functional connection between salient seed ROIs of thermal IRI and all the pixel of the face. The same approach was also applied considering as seed signal the GSR and its phasic and tonic components. Seed correlation analysis on 63 healthy volunteers demonstrated the presence of a common pathway regulating the facial thermal functionality and the electrodermal activity. The procedure was also tested on a pathological case study, finding a completely different pattern compared to the healthy cases. The method represents a promising tool in neurology, physiology and applied neurosciences.

## Introduction

Facial thermography is one of the most prevalent applications of thermal infrared imaging (IRI) in psychophysiology and medicine. The face is a naturally exposed human body area that a non-contact instrument, such as a thermal infrared camera, can easily access. The assessment of facial thermal patterns is crucial, and the literature on facial thermography is vast, ranging from thermal comfort assessment in building environments [1, 2] to psychophysiological and medical applications [3–8]. Numerous regions of interest (ROIs) have been identified in the literature as crucial for the psychophysiological characterization of a subject (i.e. nose tip and glabella ROIs).

However, investigations examining the functional link among these ROIs and about the physiological basis of the relation existing between thermal IRI and other physiological parameters (i.e. sudomotor, respiratory and cardiac activities) are still lacking.

In the past, basic research centered on the physiological elements of facial thermoregulation, examining the processes underlying perspiration and flushing [9–11]. Drummond et al. expanded the understanding of the normal physiology of face thermoregulation by focusing on flushing, perspiration, and blood flow mediated by the autonomic sympathetic nervous system (SNS) and compared it to pathologic behavior. They discovered that thermoregulatory facial flushing, emotional vasodilation, and sweating are mediated by conventional cervical sympathetic pathways [11] and those sympathetic vasoconstrictor fibers employ a tonic constrictor influence on the vasculature of the ears, lips, and nose, while sparsely supplying other parts of the face (i.e. forehead and cheeks). In addition, Drummond found that the SNS causes dilatation of the facial cutaneous vasculature in response to heat stress and emotions [9].

Facial thermal patterns result, therefore, from two distinct types of thermoregulation: emotional and physiological, which are important to be properly understood when interested to study facial temperature modulation for emotion detection.

Apart from the above-mentioned works, there is a lack in literature on the topic of functional facial thermoregulation. The central questions, therefore, are: “How is the temperature modulated by the different facial regions?” and “Does it exist a functional interlink among them?”. A possible way to answer these questions is to objectively investigate the presence of correlations between salient facial region of interest (ROIs) and all the pixel of the face. So, inspiring to the well-established brain science field using functional MRI [12–16], the authors developed a new method able to identify clusters of regions, and their correlates, that physiologically act during resting-state conditions. The primary aim of the approach was to start building foundations on the existence of a possible facial default mode network based on IRI. The hypothesis of finding a default mode network in the facial area may then contribute to a better understanding of how face reacts to emotional and non-emotional stimuli.

Furthermore, it is important to investigate the existence of a possible spatial association between facial thermal characteristics and other physiological signals, namely the galvanic skin response (GSR). The GSR has been shown to be connected with thermal IRI, and several studies have shown the feasibility of retrieving a GSR correlation from facial thermography [6, 17–19]. Evaluating the presence of a common spatial distribution of correlation between these physiological signals and temperature data could play a significant role in fundamental research as well as the investigation of the impacts of certain diseases.

If these predictions are confirmed, the implications for medical science could be profound. It could pave the way for novel uses of the technique, such as a more precise diagnosis of the spatial extent of a facial nerve injury.

The field study of facial nerve injuries is extensive, and the scientific community is well-versed in its anatomical and functional features. Many disorders, including congenital abnormalities and inflammatory, viral, and malignant syndromes, can affect the facial nerve. Bell’s palsy, for example, can cause facial nerve difficulties, tumors in the skull base or ear, infection, inflammation, stroke, and congenital disorders. Recent research [20] indicates that peripheral facial palsy can also arise during coronavirus disease (COVID-19).

Regardless of the origin, determining the precise location of nerve injury is crucial, and numerous neuroimaging techniques (e.g., magnetic resonance imaging (MRI), computed tomography (CT), and ultrasound (US)) are utilized for this purpose [21]. A less investigated aspect consists, instead, on the assessment of the peripheral effect of the facial nerve damage. Few studies concentrated on the effects of the facial nerve damage on the facial cutaneous surface using local electromyography (EMG) [22] or US but to the state of the art, there is a lack in the development of tools able to spatially identify the facial nerve damage correlates on the face. The present work aimed also at developing an innovative technique able to localize the spatial extent of the lesion of the facial surface. To this aim, together with the whole study on healthy subjects, a pathological case-study has been performed on a patient affected by congenital neuropathy.

Considering these premises, the main aim of the present work was to investigate the existence of a specific and well defined resting-state spatial pattern for the facial thermal distribution attributable to the only physiological features, not depending on a specific stimulation, being them emotional or physical. The importance of such an investigation could be that of obtaining a generalized pathway of the human face from a thermographic point of view. To the state of our knowledge this work constitutes the first attempt in this direction, combining computer vision algorithms with advanced data analysis techniques.

## Materials and methods

### Participants and experimental design

Sixty-three healthy participants (M = 25; 40% on the total sample; male to female ratio = 25:38 = 65.79%), aged 18–35 years (27.6 ± 4.8) were recruited for the experimental protocol. Exclusion criteria were to avoid any disease that may have a significant autonomic influence, including any cardiovascular, neurological, musculoskeletal, psychiatric, genetic, or congenital disease, current pregnancy or lactation, or menstrual cycle during the session. Smokers and drug addicts were excluded. Participants were asked to abstain from alcohol, caffeine, and cardiovascular exercise for 24 h before the experimental session to control for external confounds. The participants were asked to avoid the use of make-up and/or moisturizers before the experiment. Furthermore, male participants were asked to shave 48 h before the experimental session.

Volunteers from different universities were recruited by email, telephone, or direct contact. Participation in the study was voluntary, and participants were not compensated. The study was approved by the local ethics committee, and written informed consent from all participants was obtained before the experiment, according to the Declaration of Helsinki (Protocol number 15, date: 17.06.2021).

Before testing, each participant was left in the testing room for 15 min to stabilize their basal skin temperature. According to standard IRI study guidelines, the experimental room was designed as a thermoneutral environment, i.e., with a standardized temperature (23 °C) and humidity (50–60%) controlled by a thermostat to avoid thermoregulatory-induced changes [23].

### Data acquisition and analysis

During the measurement period, the facial thermal IRI and the galvanic skin response (GSR) were acquired for five minutes. Participants were asked to lay relaxed on a plinth during the measurement. The procedure for the analysis of each of the signals has been reported in separate sections below.

#### Thermal imaging data processing

A FLIR A655sc thermal camera (640 480 bolometer focal plane array (FPA), noise equivalent temperature difference (NETD) of 50 mK at 30 °C) was used to capture facial thermal IRI. Concurrently, a visible video was recorded using a webcam (Logitech© C920 HD PRO (1080 × 1920 pixels, full HD lens)). Visible imagery was employed to track 68 facial landmarks through the software OpenFace [24], and, successively, they were co-registered to the IRI by the estimation of the geometrical transformation between the visible and the IR optics, following the same procedure described in [25]. Both the videos were acquired at a frequency rate of 10 Hz, thus allowing for time synchronization. The estimated facial landmark locations on IRI were then employed to warp thermal images on a reference template by means of a Local Weighted Mean (LWM) geometrical transformation [26]. With such a procedure, it has been possible to rely for all the subjects on a common reference for the location of facial areas in terms of pixels, thus allowing for a successively accurate and objective comparison among subjects.

**Fig. 1:**
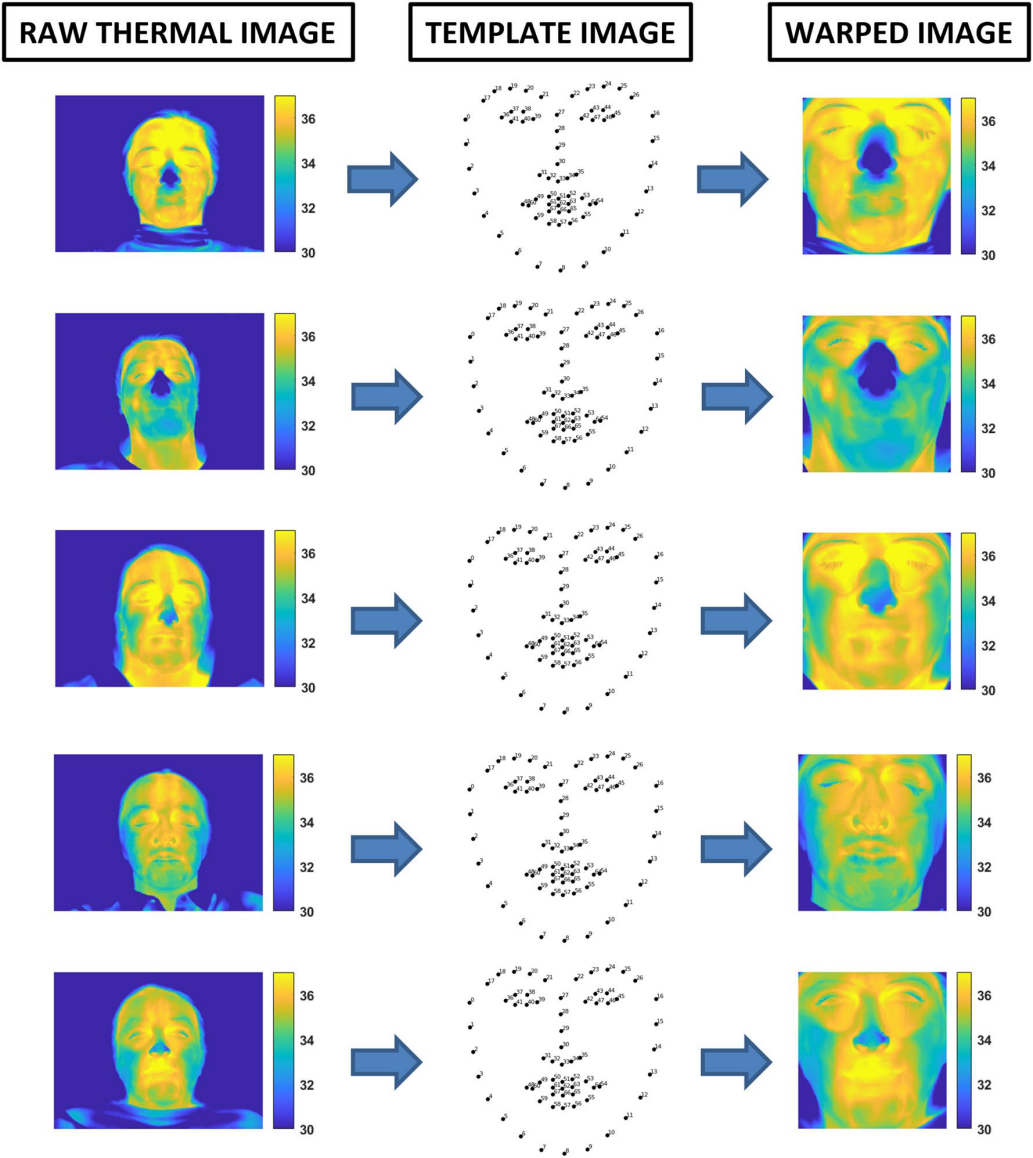
Thermal IRI processing scheme. The raw thermal images of the participants were warped on the template to obtain the warped images. The adopted procedure is described in [25]

An example of the thermal IRI processing is reported in Fig. 1.

The entire warping procedure is automated, and no intervention by a human user is required. This ensures a completely user-independent analysis, thus overcoming the bias due to classical IRI processing approaches in which the user defines the ROIs to study, usually manually selecting the areas to consider for further analyses. The procedure has already been validated, and two technical publications have deepened the understanding of the method’s performance [25, 27]. In particular, the algorithm described in Cardone, 2021 reported a good spatial accuracy (average Root Mean Square Error, RMSE = 0.66 pixel) as well as a good temporal accuracy (RMSE = 0.81 ms) relatively to the co-registration between visible and IR imagery. This performance ensures reliable estimation of regional temperature (RMSE = 0.09 °C) within a defined range of head rotation (± 24.23° for yaw and ± 13.79° for pitch movements), thus revealing to be robust to inter-subject facial geometrical and anatomical differences and intra-subject movement-related noise. Furthermore, the technique has been employed in several studies [28–30].

For the present study, each of the thermal image of each subject was warped onto the common template, usually used in computer vision application for face detection and recognition [25, 31–35]. This allowed to rely on an average of 3000 warped images per subject.

#### Galvanic skin response processing

The GSR was recorded on the thenar/hypothenar muscles of the non-dominant hand [36] by means of the AD instrument Powerlab system, featured by a low-voltage GSR amplifier, 75-Hz AC excitation, and automatic zeroing. The finger electrodes were made of stainless steel and held with Velcro tape. The sampling frequency was set to 1 kHz. The GSR signal was filtered with a zero-lag third-order Butterworth bandpass filter (0.01–5 Hz) [6] and then down-sampled to 10 Hz, to be homogenized with the IRI. The tonic and phasic components of the signal were separated using a continuous decomposition analysis provided by Ledalab, which is Matlab-based software [37].

### Statistical analysis

To assess the spatial correlation of the single seed ROI (sROI) in the thermal IRI context and also with respect to the GSR signals, a seed correlation analysis (SCA) approach was developed [38, 39].

**Fig. 2:**
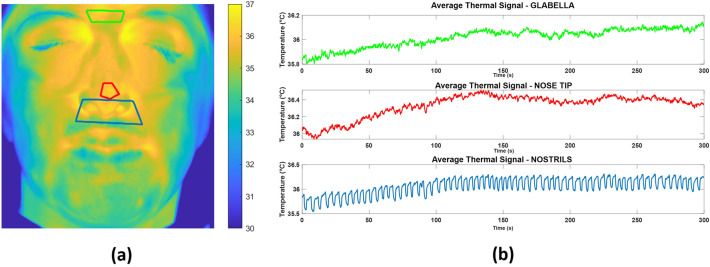
Average temperature signals extracted over the three seed ROIs: **a** ROI seeds locations (GLABELLA in green, NOSE TIP in red, NOSTRILS in blue); **b** thermal signals extracted over time in the seed ROIs

In particular, according to the relevance of the ROIs, as reported by the scientific literature [40], three different seed regions were chosen: the nose tip (NT), the nostrils (NOSTR) and the glabella (GL) ROIs (Fig. 2a). For each one of these ROIs, the average temperature was evaluated over all the pixels in the ROI, and the temporal dynamic of the signal was extracted for all the frames of the video, thus obtaining a thermal signal such as the one reported in Fig. 2b.

In particular, for each ROI:

1 $$\overline{{{\varvec{T}}_{{{\varvec{sROI}}}} }} \left( {\varvec{n}} \right) = \frac{1}{{\varvec{N}}}\mathop \sum \limits_{{{\varvec{p}} = 1}}^{{\varvec{N}}} {\varvec{T}}\left( {{\varvec{x}}\left( {\varvec{p}} \right),{\varvec{y}}\left( {\varvec{p}} \right)} \right)$$

where ${\varvec{T}}_{{{\varvec{sROI}}}}$ is in turn the average temperature signal relative to *NT, NOSTR, GL*, ${\varvec{n}}$ is the number of frames of the video, ${\varvec{T}}\left( {{\varvec{x}}\left( {\varvec{p}} \right),{\varvec{y}}\left( {\varvec{p}} \right)} \right)$ is the temperature value in the pixel with coordinates $\left( {{\varvec{x}}\left( {\varvec{p}} \right),{\varvec{y}}\left( {\varvec{p}} \right)} \right)$ and ${\varvec{N}}$ is the total number of pixel in the ROI under consideration.

**Fig. 3:**
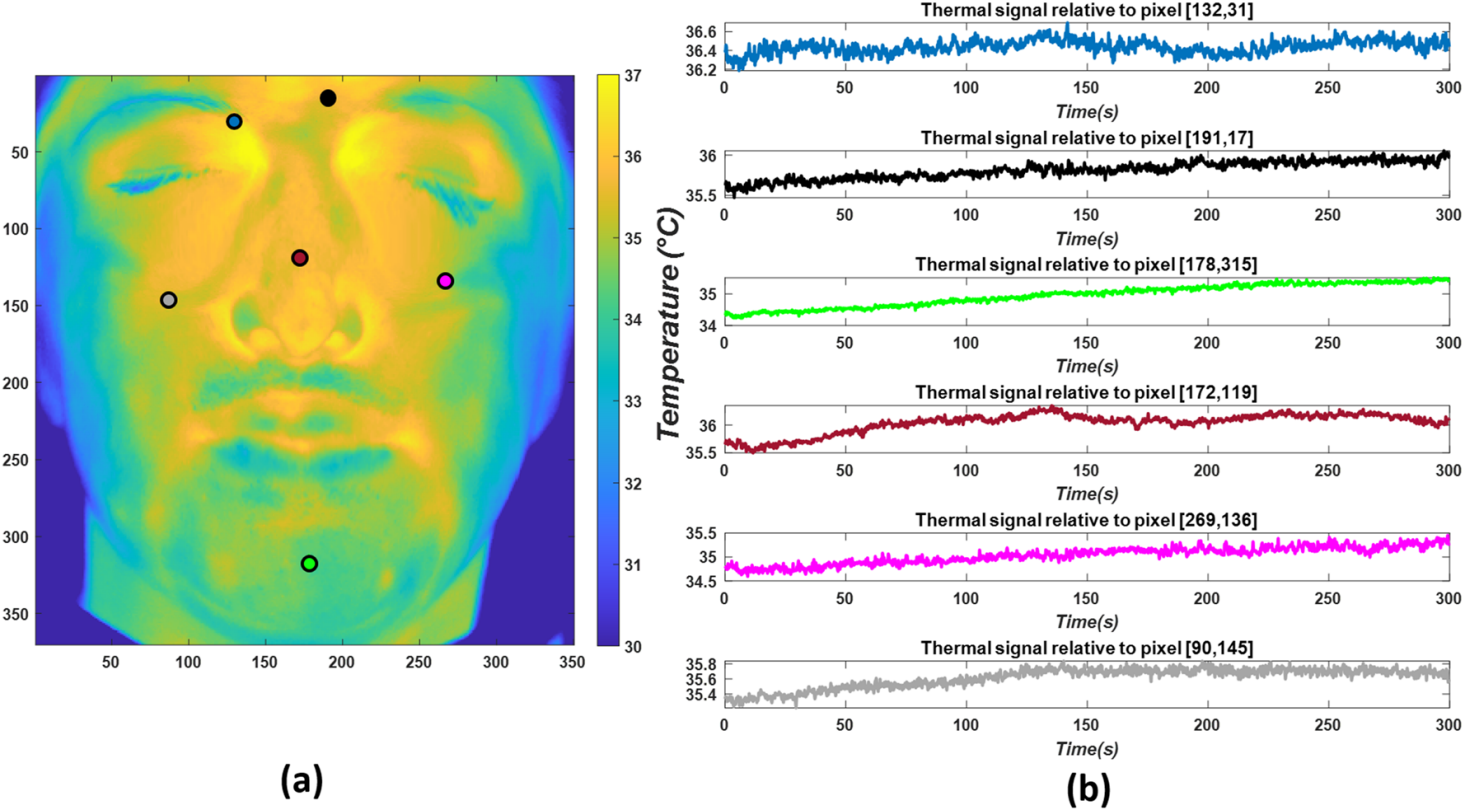
Average temperature signals extracted over six pixels of the face: **a** pixel location; **b** thermal signal relative to the pixel highlighted in **a** with corresponding colors

The thermal signal relative to each pixel ${\varvec{T}}\left( {{\varvec{x}}\left( {\varvec{p}} \right),{\varvec{y}}\left( {\varvec{p}} \right)} \right)$ was extracted over time. Figure 3 represents thermal signals obtained from different pixels, relative to a given subject.

**Fig. 4:**
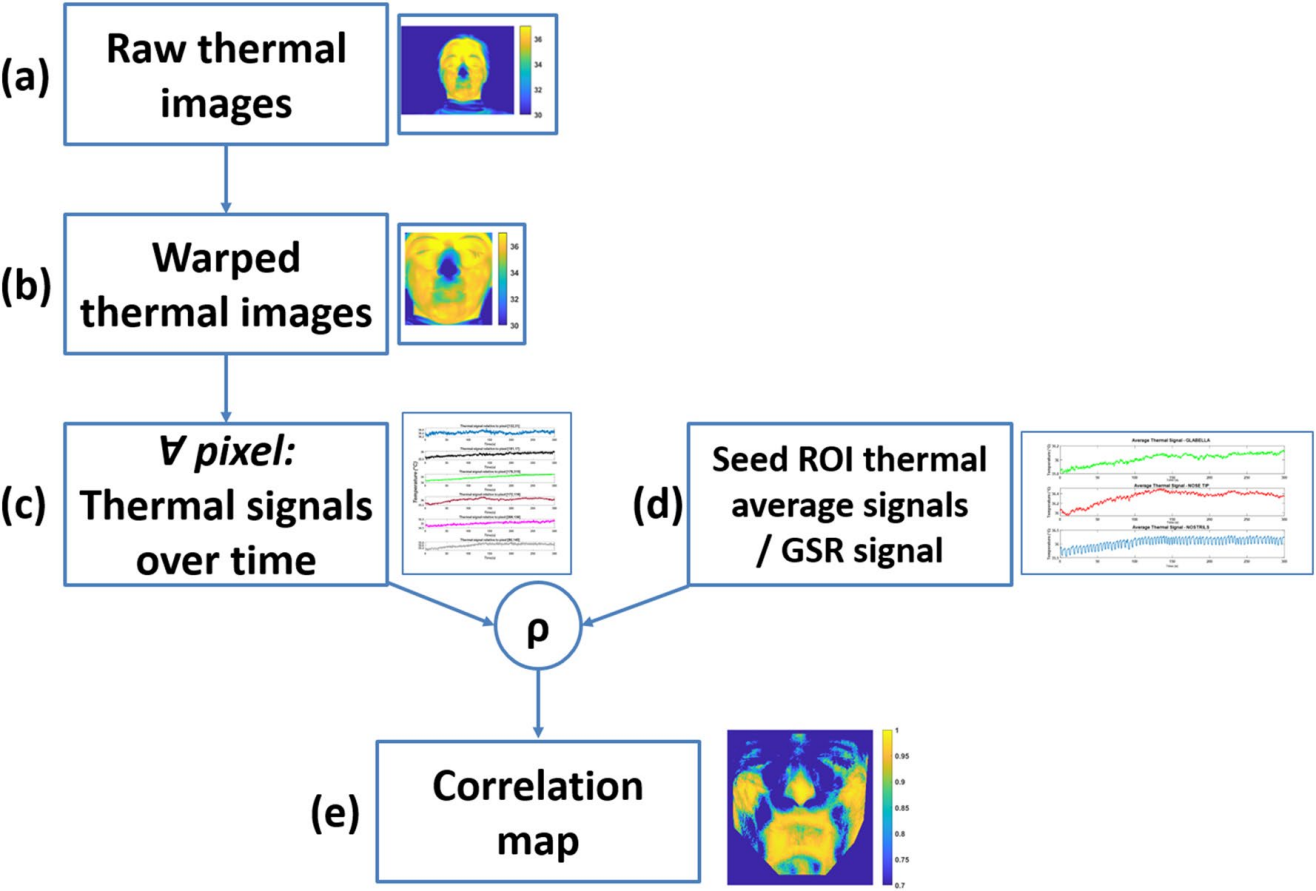
Procedure of analysis applied in the present work. The raw thermal images (**a**) of the subject are warped on a template, thus obtaining the warped thermal images (**b**). Thermal signals are extracted over time for each pixel (**c**) from the warped thermal images. A correlation is computed between each of the extracted thermal signal (**c**) and the seed ROI/GSR signals (**d**). A correlation map is thus obtained for each participant (**e**)

For each seed ROI, the correlation between $\overline{{{\varvec{T}}_{{{\varvec{sROI}}}} }} \left( {\varvec{n}} \right)$ and the temporal thermal signal relative to each pixel ${\varvec{T}}\left( {{\varvec{x}}\left( {\varvec{p}} \right),{\varvec{y}}\left( {\varvec{p}} \right)} \right)$ was computed, thus obtaining for each subject a correlation map (Fig. 4). The pixel related to the eyes region were excluded from the analyses due to the movement artifact following blinking.

It is worth to note that thanks to the application of the warping procedure on the whole images of the dataset on a common template (“Thermal imaging data processing” section), the ROIs positions will be the same for the different subjects, as well as for the single case patient.

The same approach was also adopted for the seed correlation map relative to the GSR signal. In this case, the raw GSR signal, the tonic component (GSR-T), and the phasic component (GSR-P) were used as seed signals for the computation of the seed correlation maps. Figure 4 depicts the entire procedure of analysis applied in the present work for both spheres of interest, i.e., thermal signal seeds and GSR related seeds.

The average values of the correlation maps were computed for all the subjects, thus obtaining a global seed correlation map. Standard errors of the correlation maps were also computed.

Cluster analysis by means of k-means algorithms [41] was applied to the average seed correlation maps to automatically identify salient patterns. K-means clustering is a method of data grouping that aims to partition *n* observations into *k* clusters. Each observation belongs to the cluster that has the closest mean, or cluster centroid, acting as the cluster’s model. By employing particular metrics, k-means clustering reduces within-cluster variations. In the case of this study, a *city-block* metric was selected. The *city-block* metric relies on the sum of absolute differences [42]. Each centroid is the component-wise median of the points in that cluster.

All the above mentioned analyses were performed on the 63 healthy subjects. The same method was used for the single case-study patient.

## Results

### Performance of the image processing system

With regard to the quality of the combined visible-IR imaging tracking procedure, on average 98,84% of the video frames were correctly processed, whereas the face classification index, revealed as output by the OpenFace software, was 0.95 (on a maximum value of 1). These findings highlight the developed tracker’s performance and provide a solid foundation for subsequent analyses.

Considering the goodness of the warping procedure, relying on the results obtained in previous work by the authors, a LWM geometrical transformation was employed to warp each image on the common template, thus ensuring the least amount of error possible due to the warping procedure [25]. As reported by Cardone and colleagues, the method allows for both spatial (Root Means Square Error (RMSE) = 0.66 pixel) and temporal (RMSE = 0.81 ms) accuracy.

### Seed correlation maps for healthy subjects

**Fig. 5:**
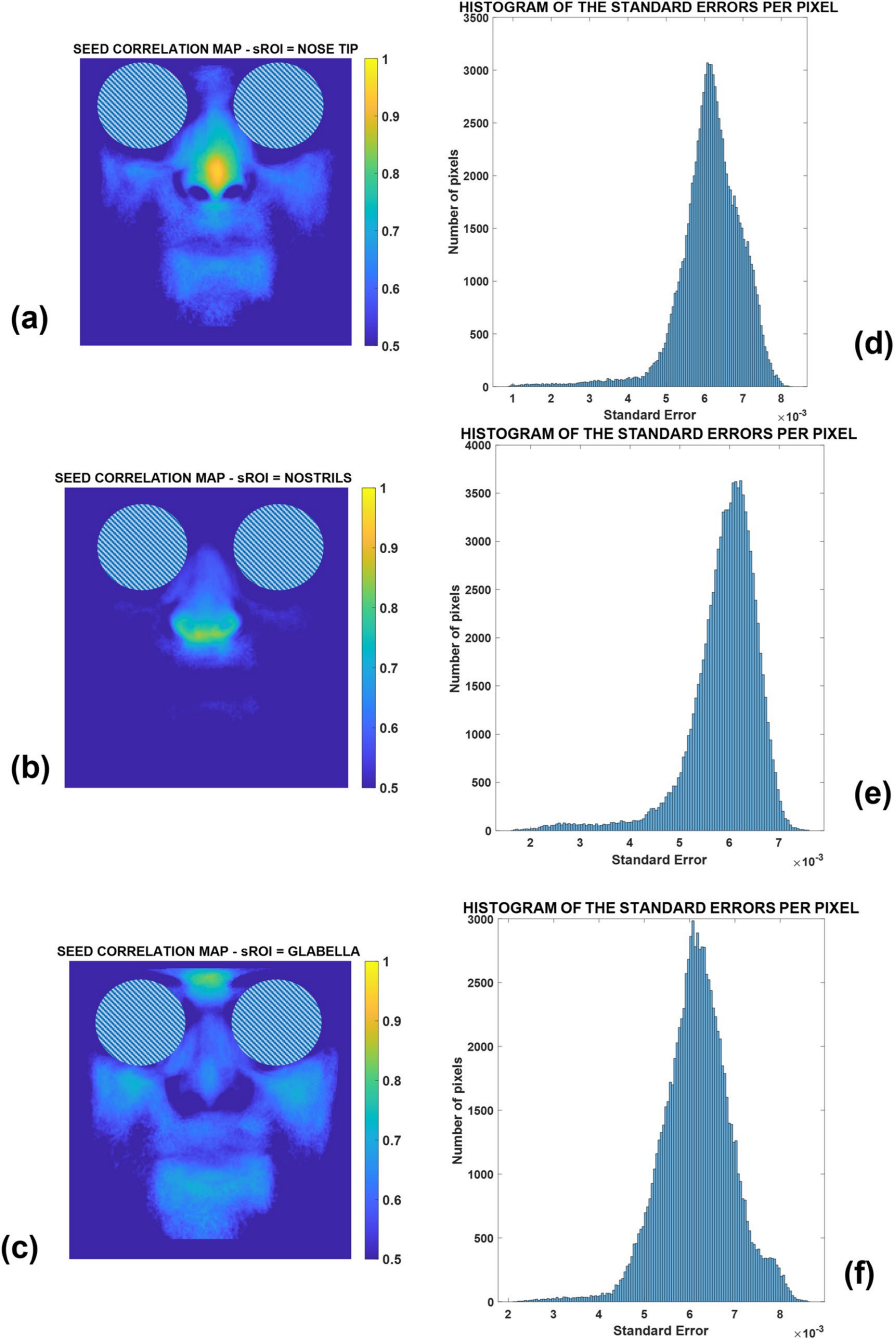
Seed correlation maps relative to thermal seeds ROIs for: **a** nose tip seed, **b** nostrils seed, **c** glabella seed. Histograms of the standard errors per pixel are shown for the values of correlation related to **d** nose tip seed, **e** nostrils seed, **f** glabella seed

The results of the average correlation maps computed, i.e., the global seed correlation maps, are reported in Fig. 5 for the thermal ROI seeds of *NT* (Fig. 5a)*, NOSTR* (Fig. 5b)*,* and *GL* (Fig. 5c). Standard errors of the correlation maps are also reported in Fig. 5d–f.

It is clearly possible to observe that the maximum values of the correlation for each map are around the seed location. Another relevant finding is related to the specific patterns of the correlation maps. It can be observed that the patterns relative to the NT and GL are similar, whereas the pattern relative to the NOSTR is unique, thus demonstrating that the effect of the breathing signal is not widespread over the face, resulting in limited spatial extension (i.e., the most correlated regions concern the nose and the immediate lower perioral area). Furthermore, referring to the distributions of the standard errors (Fig. 5d–f), it is clearly noticeable that the results are very reliable, given the low level of the mean standard errors (Table 1).

**Table 1:** Mean standard error for each case of analysis

SEED	Average standard error (× 10^–3^)
Nose tip (NT)	6.1
Nostrils (NOSTR)	5.9
Glabella (GL)	6.2
GSR	8.1
GSR-phasic component (GSR-P)	2.8
GSR-tonic component (GSR-T)	7.3

The average has been performed among all the subjects and for all the pixels

**Fig. 6:**
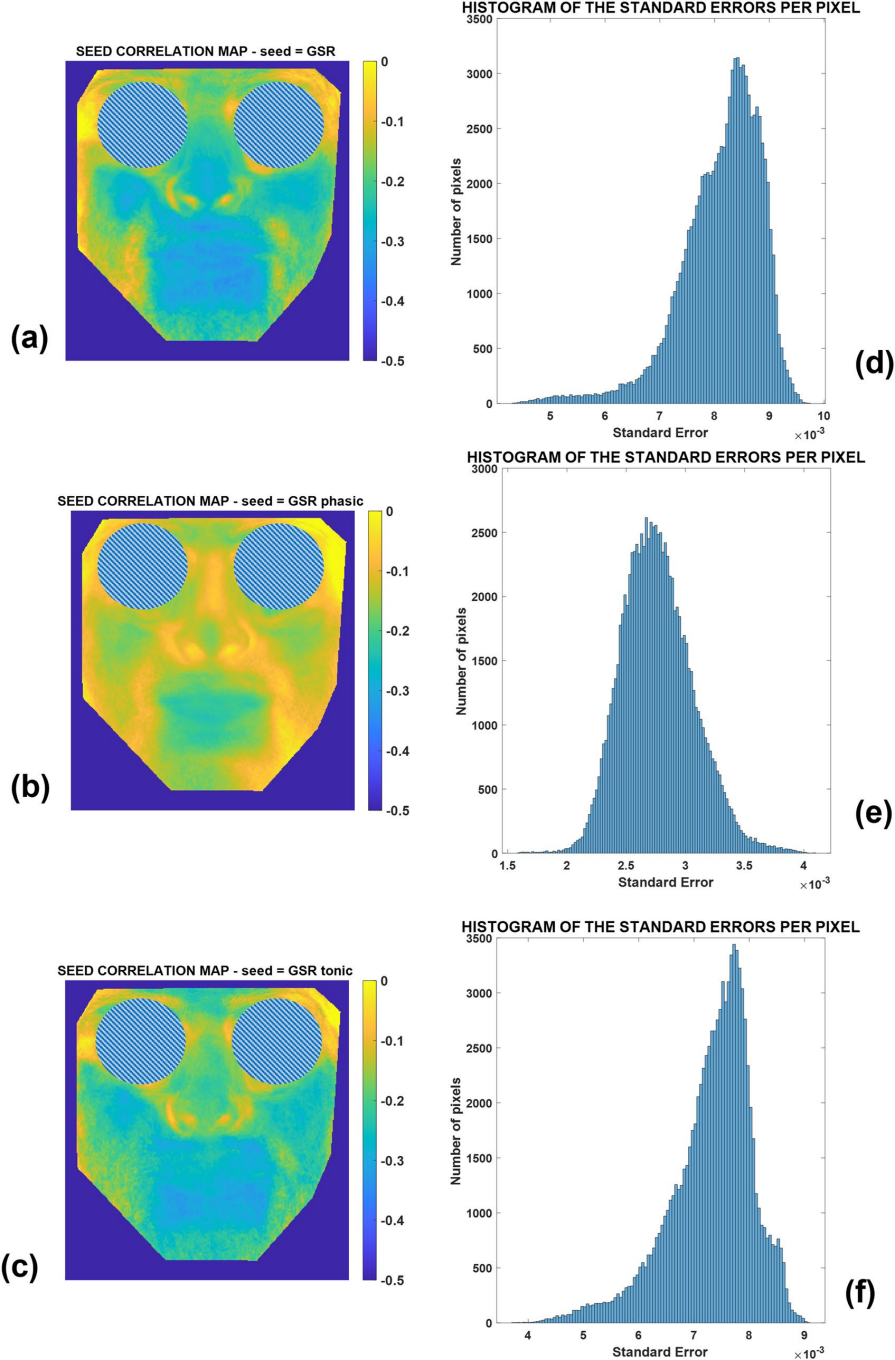
Seed correlation maps relative to the GSR seeds and specifically for: **a** the whole GSR seed, **b** the phasic component of the GSR seed, **c** the tonic component of the GSR seed. Histograms of the standard errors per pixel are shown for the values of correlation related to **d** the whole GSR seed, **e** the phasic component of the GSR seed, **f** the tonic component of the GSR seed

Figure 6 instead represents the average correlation maps for the GSR (Fig. 6a), GSR-P (Fig. 6b), and GSR-T (Fig. 6c) seeds. Figure 6d–f show the standard errors of the correlation maps.

It is possible to notice that when the seed is the GSR or its component, the correlation map shows negative values, meaning that the GSR signal is anti-correlated with respect to the majority of the thermal signals of the facial pixels. This is a common pattern for the whole GSR signal and the tonic component in particular, thus meaning that the major contribution to the correlation map relative to the whole GSR is given by the tonic component, rather than the phasic component. In fact, the correlation map relative to the phasic component shows general values closer to zero compared to the map relative to GSR-T. In the case of the GSR, it is also clear that the results are very reliable, as evidenced by the low level of mean standard errors (Table 1).

Table 1 summarizes the mean standard error for each case of analysis. The values are averaged for each pixel, and taking into account all the participants.

**Fig. 7:**
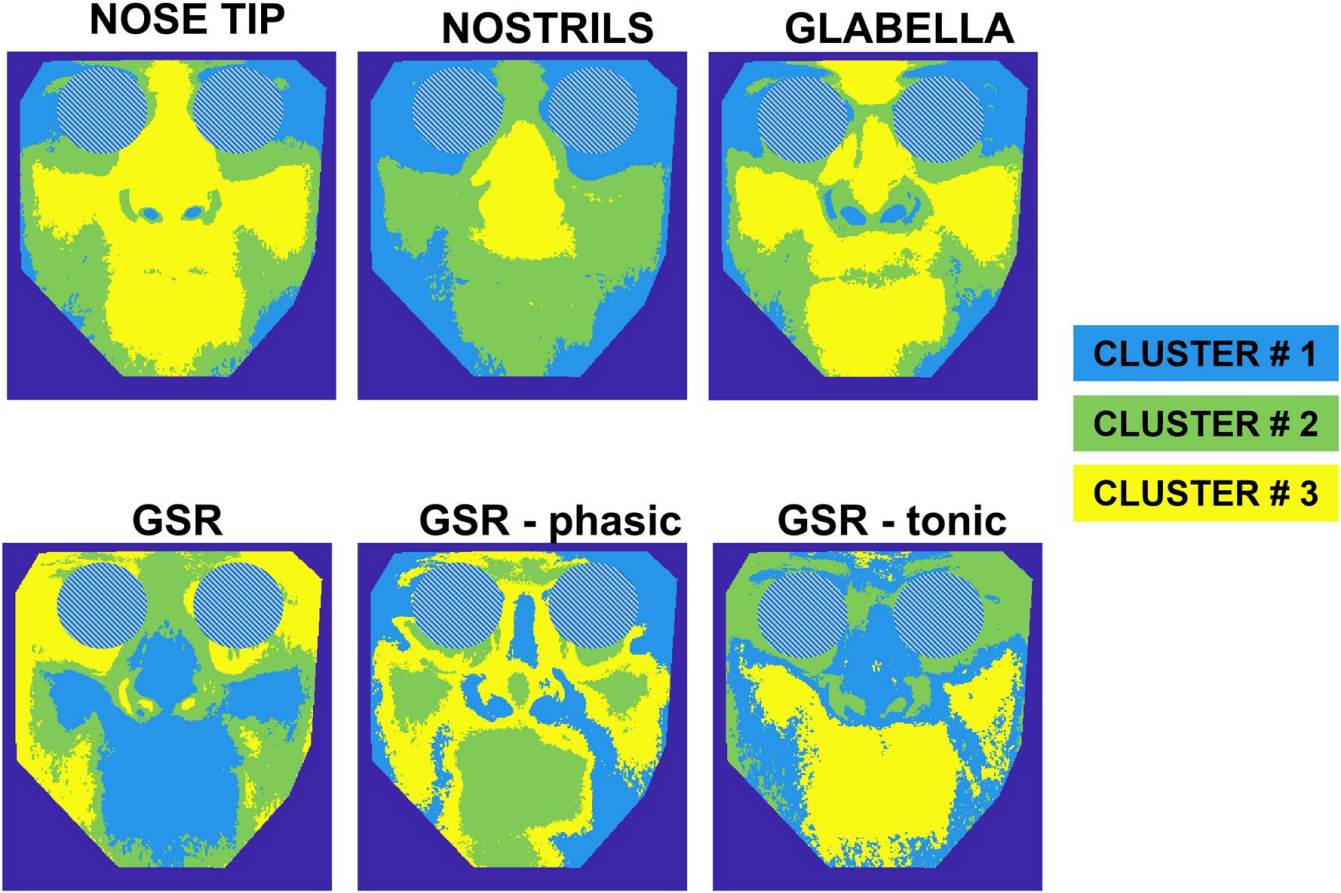
Results of the k-means cluster analyses relying on the correlation maps of nose tip, nostrils, glabella, the whole GSR, GSR-P, GSR-T seeds. Numerical values of the clustering are reported in Table 2

Figure 7 represents the results of k-means cluster analysis with k = 3. This means that the correlation maps were clustered into three groups. The results of the k-means cluster are reported in Table 2 for each specific case of analysis.

**Table 2:** Results of cluster analyses for each case

SEED	Number of iteration	Cluster centroids	Mean distance per cluster	Within-cluster sum of distances
Nose tip (NT)	38	0.34	0.18	805.97
		0.46	0.10	1021.89
		0.59	0.12	2143.23
Nostrils (NOSTR)	20	0.34	0.11	1865.18
		0.46	0.09	1197.16
		0.59	0.19	723.28
Glabella (GL)	22	0.39	0.16	994.88
		0.51	0.09	926.22
		0.62	0.10	1371.76
GSR	27	− 0.18	0.06	644.87
		− 0.12	0.08	756.99
		− 0.27	0.09	760.65
GSR-phasic component (GSR-P)	24	− 0.07	0.06	623.74
		− 0.12	0.04	409.34
		− 0.17	0.05	657.67
GSR-tonic component (GSR-T)	42	− 0.19	0.05	568.81
		− 0.12	0.08	811.62
		− 0.26	0.08	661.90

Number of iteration for convergence, cluster centroids, mean distance per cluster and within-cluster sums of point-to-centroid distances are reported below

It is worth noting that, although the distribution of correlation values for the GSR-seed maps is in the opposite direction compared to the case of thermal seeds, the spatial distribution of temperature clusters is roughly the same, with a distribution of the areas mostly correlated creating a “butterfly” pattern, which includes the glabella, perioral area, nose and cheeks. This “butterfly” pattern is commonly present in all the seed correlation maps apart from the only case of the nostrils seed, meaning that the respiratory signal does not impact the whole thermal pattern of the face, affecting only the lower nasal and upper lip regions (Figs. 5b, 7).

### Seed correlation maps for case-study patient

**Fig. 8:**
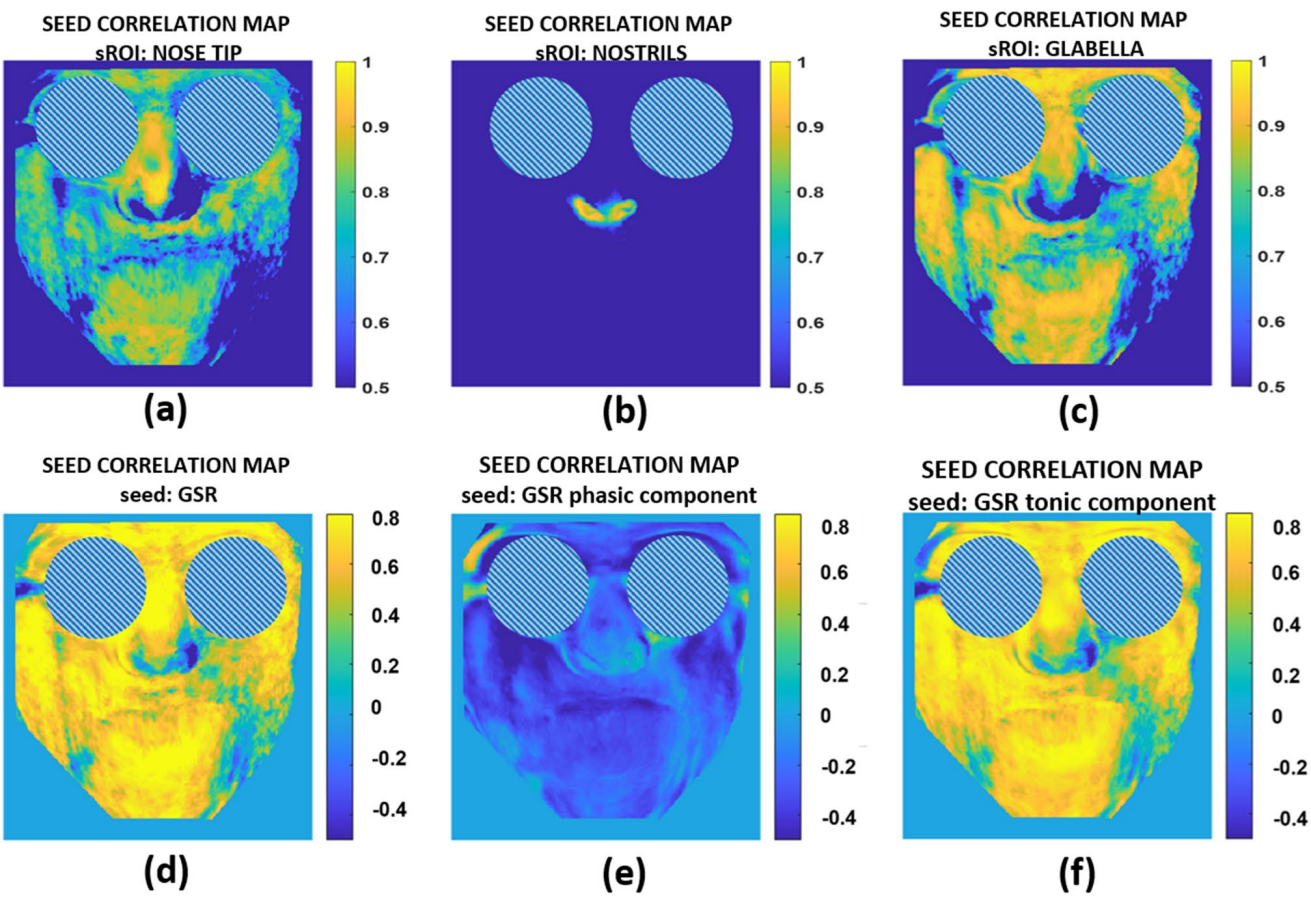
Seed correlation maps relative to thermal seeds ROIs for: **a** nose tip, **b** nostrils, **c** glabella, **d** the whole GSR, **e** the phasic component of the GSR, **f** the tonic component of the GSR seeds

The same procedure of analysis described in the Methods section was applied to the signals acquired from the patient. Figure 8 shows the seed correlation maps for the thermal seed (NT, NOSTR, GL) as well as the GSR signal and its components (GSR-P, GSR-T).

It is clearly observable that the seed correlation map relative to the nostrils (Fig. 8b) is very similar to that of the healthy subjects (Fig. 5b), being the lower part of the nose being the only region involved in the correlation. All the other correlation maps are very dissimilar compared to the healthy ones. The “butterfly” pattern is not present, instead, it is possible to clearly identify the limits of the functional lesion of the patient (Fig. 8a, c, d, f, bottom right side of the image, bottom left side for the patient). The nasal tip, glabella, and GSR and GSR-T related maps all show the spatial pattern of the patient’s dysfunction.

**Fig. 9:**
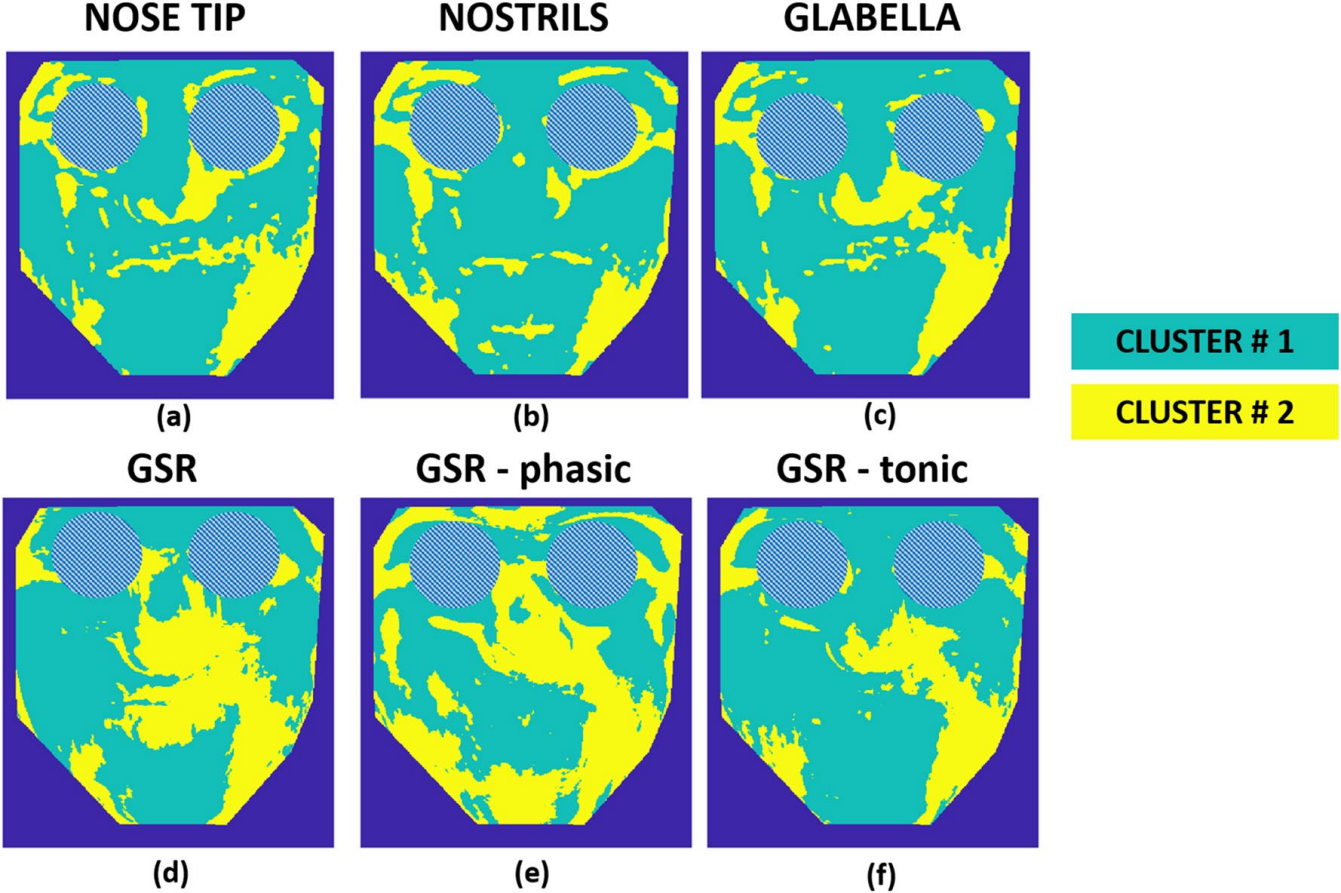
Results of the k-means cluster analyses relying on the correlation maps of nose tip, nostrils, glabella, the whole GSR, GSR-P, GSR-T seeds for the case-study patient. Numerical values of the clustering are reported in Table 3

Additionally, only for explorative purposes, the k-means clustering algorithm with k = 2 was applied to the correlation maps of the patient. Results are shown in Fig. 9. The output of the k-means clustering, with the specific metrics, is reported in Table 3.

**Table 3:** Results of cluster analyses the patient case-study

SEED	Number of iteration	Cluster centroids	Mean distance per cluster	Within-cluster sum of distances
Nose tip (NT)	22	0.78	0.14	3593.03
		0.53	0.22	4376.54
Nostrils (NOSTR)	22	0.37	0.05	2291.15
		0.29	0.08	1274.02
Glabella (GL)		0.87	0.13	3254.70
		0.62	0.21	4055.40
GSR	22	0.78	0.14	3593.03
		0.53	0.22	4376.54
GSR-phasic component (GSR-P)	27	− 0.38	0.15	3896.48
		− 0.17	0.17	4399.57
GSR-tonic component (GSR-T)	13	0.70	0.16	4661.06
		0.29	0.34	3788.41

Number of iteration for convergence, cluster centroids, mean distance per cluster and within-cluster sums of point-to-centroid distances are reported below

It is possible to observe that the boundary of the lesion on the patient can be automatically identified, through a completely automatic and objective procedure, underlying the importance of the developed approach for diagnostic purposes. It is worth noticing that the algorithm has been applied only to one case-study, which clearly depicts the importance of the method if tested on a wider cohort of patients.

To summarize, from the presented results, it is possible to observe that the thermal seeds located on the nose tip and glabella (the area between the eyebrows) exhibit a precise and distinctive association with the pixels on the face, forming a butterfly-shaped pattern. When studying the correlation between Galvanic Skin Response (GSR) and its tonic component with all the thermal signals of the facial IRI, the same pattern was consistently obtained. These findings provide evidence for the existence of a functional pathway that influences the described behavior. Additionally, the results were reinforced by examining a pathological case study, which showed correlation maps with an asymmetrical pattern for each of the seed correlation maps, instead of the “butterfly” structure observed in the other cases. To the state of our knowledge, this is the first time that this kind of approach has been developed in the thermal IRI area of research.

## Discussion

This study presents a new methodology for facial thermal IRI to evaluate the spatial and functional correlation patterns between average thermal signals collected from salient seeds (i.e., nose tip, nostrils, glabella) and the temperature distribution over the entire facial pixels. IRI, together with a physiological signal, i.e. the GSR signal, were recorded for 5 min during rest in 63 healthy subjects. Seed correlation maps were generated by establishing a pixel-based correlation between the seeds (IRI or GSR-based) and the temperature signals of each pixel. To the author’s knowledge, this paper marks the first attempt to examine these functional correlations using computer vision algorithms with advanced data analysis tools.

The entire technique is based on a warping approach that the authors developed and validated [25, 27]. Sixty-eight facial landmarks tracked by the software OpenFace on the visible videos served as the foundation for identifying the features points in thermal IR imaging. A warping algorithm was applied to IRI based on the locations of facial landmarks, resulting in a series of images for each subject warped on the same template (Fig. 1). Correlation maps were then obtained on a pixel basis between each pixel thermal signal and thermal seeds or GSR signals.

The resulting correlation maps (Fig. 5) showed specific, consistent and repeatable patterns. Indeed, the correlation map based on the nose tip and the glabella seeds displayed a pattern resembling a “butterfly” shape, as shown in Figs. 7a and c, which represents the output of the k-means clustering method with k = 3. In contrast, the correlation map relative to the seed of the nostrils, and thus solely related to the respiratory activity, did not display this distinct and unique pattern, emphasizing the selected area responding to respiratory function (Fig. 5b). Indeed, only the region immediately surrounding the nostrils, the nose, and the top portion of the perioral region were substantially connected to the respiratory seed signal, as demonstrated by the k-means clustering outcome (in yellow in Fig. 7b).

Considering possible physiological mechanisms of action, one main element might be responsible of the observed outcomes: the sympathetic vasomotor and sudomotor fibers (SVSF), which are post-ganglionic fibers originating from the superior cervical ganglia and receiving information from the second to the forth thoracic vertebra (T2–T4) preganglionic sympathetic fibers [9, 11, 43]. These SVSF innervate the whole face with topographical distribution towards the forehead, cheeks (maxillary area) and perioral area controlling the face temperature via eccrine sweat gland stimulation (sudomotor) and vasoconstriction (vasomotor). Indeed, the SVSF are related to the vascular system, specifically to the internal and external carotid artery. The former is associated to the SVSF, receiving presynamptic fibers from T2, that innervate the middle part of the forehead; whereas the latter to SVSF from T3 and T4, that innervate the maxillary and perioral area. This system is demonstrated to be primarily responsible for facial blood vessel constriction and dilation [11, 44]. The sympathetic pathway can, therefore, promote eccrine sweat production and vasodilation in the skin to adapt the body temperature through the sudomotor and vasomotor axon reflex. Yet, current research reveals a vast diversity in axon reflex variability. Local iontophoresis of cholinergic agonists, such as acetylcholine, can stimulate the sudomotor axon reflex. These molecules bind on muscarinic receptors of the sweat gland to induce direct sweating locally and bind on nicotinic cholinergic receptors on postganglionic sympathetic C-fiber terminals to trigger an antidromic impulse along the postganglionic sympathetic C-fiber. At the branching points of this fiber, it transforms into an orthodromic impulse that stimulates nearby eccrine sweat glands via the indirect axon reflex-mediated release of cholinergic agent [45–49]. Non-sympathetic afferent C- and A-fibers can also cause an axon reflex. Iontophoresis of cholinergic agents directly vasodilates and depolarizes non-sympathetic sensory nociceptive A- and C-fibers and sympathetic C-fibers to trigger the vasomotor axon reflex. The nerve impulse travels orthodromically to a branching point, unlike the sudomotor axon reflex. This branching point conducts antidromically towards a blood vessel terminal bouton. Axon reflex-mediated vasodilation results from vasoactive neuropeptide release. Adrenergic molecules can locally produce vasoconstriction [45, 50–52].

Another possible mechanism might involve the trigemino-parasympathic complex, which is responsible of the parasympathetic control of the face. Of particular interest for the thermoregulation purpose of this discussion is the diving reflex that seems to have a possible role. Indeed, it is a reflex that can causes bradycardia, apnea, and increased peripheral vascular resistance due to a stimulation of the trigeminal nerve. As a consequence of combined face stimulation and apnea, this extremely powerful cardiovascular reflex response occurs by decreasing the sympathetic conductivity toward the cutaneous vascular beds, which have a larger role in thermoregulation and sweating.

In addition to the seed correlation analysis based on thermal seeds, GSR signals including the tonic and phasic components were used to generate additional seed correlation maps. Several scientific investigations have already proven a close association between the GSR signals, their components, and the facial heat signals derived from salient regions of interest (i.e. nose tip, glabella) [6, 19, 53]. GSR and thermal signals are typically anti-correlated since when the GSR signal (i.e., sudomotor activity) increases, the temperature of the implicated ROIs falls due to the cooling impact of sweat. This study, however, extended previous work by clarifying and deepening this specific link, highlighting the spatial and functional pattern. As shown in Fig. 6a, the “butterfly” pattern is again repeated confirming the consistency of the phenomena demonstrated on the thermal seeds of the nose tip and glabella. The k-means cluster analysis supports this conclusion by highlighting the unique cluster of pixels with a shared behavior (Fig. 7d).

Regarding the tonic and phasic components, the former should predominate over the latter in resting-state situation such as that of this research. It is worth noting that the tonic component is elicited in reaction to external inputs such as auditory, tactile, visual, or emotional stimuli [34, 35, 54]. Focusing on Fig. 6b and c, one can note that the absolute amplitude of the correlations is smaller for the GSR-P correlation map than the GSR-T correlation map. In addition, the pattern revealed by the cluster analysis of the seed correlation map of GSR-T (Fig. 7f) overlaps significantly with that of the entire GSR (Fig. 7d). Notably, clustering applied to the seed correlation map relative to the GSR-P revealed a pattern that could approximate the “butterfly” shape. However, it is essential to highlight that this result is derived from a correlation map with a lower order magnitude compared to the entire GSR and GSR-T maps. This conclusion is further supported by the examination of the cluster centroids presented in Table 2, which reveals that the absolute values of the GSR-P cluster centroids are lower than those of the GSR and GSR-T cluster centroids.

The common “butterfly” shape identified on both the thermal and GSR seeds-related correlation maps confirms the significance of the sympathetic and trigemino-parasympathetic pathway in regulating the thermal response of the face.

Relating to the case-study, a patient with a facial nerve deficiency gave the opportunity to test the procedure in a clinically-based context. Except for the seed correlation map for the nostrils, it is evident that there is a major difference between each of the case-study maps and the corresponding ones of the healthy subjects. The “butterfly” pattern observed in healthy persons is absent showing asymmetric patterns. Like in healthy subjects, clustering analysis (k = 2) reveals a high degree of similarity between the nose tip, glabella, GSR, and GSR-T maps. (Fig. 8a, c, d, f). The most notable finding in this case is that the subject’s area of impairment is easily identified (left bottom of the face, i.e. right bottom in the image of Fig. 9a, c, d, f).

However, since the results are based on data from a single patient, no statistically significant inferences can be reached; however, the findings are noteworthy and pave the way for more in-depth investigations that permit generalization to other facial nerve disorders. One of the future developments of this area of research will be enrolling more patients with various diseases (such as harlequin syndrome, facial hemiparesis, hemicrania, Moebius syndrome) and apply the developed methodology. Patient-based studies could lead to important knowledge about these specific diseases and open the way to developing specific diagnostic tools for clinicians. The ability to identify the precise area of functional impairment in the facial surface could be of great value to clinicians since the technique could also be used to monitor the efficacy of therapy over time. In this regard, the method could be utilized as a follow-up tool, with the capacity to estimate the number of pixels effectively implicated in the pathology, including sympathetic deficiency, and be beneficial for determining a treatment’s efficacy. Another potential future advancement would be to reproduce the study using additional physiological signals, such as the electrocardiogram or photoplethysmography. A number of researches have established a robust connection between these two types of physiological signals and facial IRI [55, 56].

Moreover, further studies should also be performed to investigate whether there is an effect on the presented results due to the age and gender of the participants. A larger sample needs to be enrolled in future studies involving both males and females in a larger cohort and also across different age ranges from infant to geriatric subjects.

The work is original, influenced by established fMRI analysis techniques, and may constitute a medical breakthrough. The primary objective of this study was, in fact, to examine the presence of a distinct and clearly defined spatial pattern in the thermal distribution of the face during a resting state. This pattern was sought to be independent of any specific stimulation, whether emotional or physical, and solely attributed to physiological characteristics. The significance of this investigation lies in obtaining a comprehensive understanding of the thermographic perspective of the human face. To the best of our knowledge, this study represents the first endeavor in this direction by combining computer vision algorithms with advanced data analysis techniques. The established method provided a better understanding of the functional link among thermal areas over the face on a pixel-by-pixel basis. With respect to the previously conducted physiological research [40, 57, 58], relying on examining the temporal dynamics of facial thermal signals, for the first time ever we were able to deepen the interrelationship of the thermal signals from a pixel-by-pixel perspective. Obtaining a concept of the effective area of impairment in neurological pathology is also crucial for clinical purposes, but no technology currently provides this information. Indeed, only invasive, complex and even costly exams like Laser doppler flowmetry, two-dimensional Laser doppler imaging, and axon-reflex based tests of sudomotor and pilomotor function, such as the quantitative sudomotor axon reflex test, can be used to evaluate the functional integrity of the SVSF. Conversely, IRI is contactless, non-invasive, and no energy dose is administered to the patient, making it a very useful and promising imaging technique in clinical settings. Lastly, it should be emphasized that although the established method has been used to facial IRI, it can be fully expanded to the study of the functionality of other body regions, allowing for the estimation of the true degree of a functionally impaired area.

## Conclusions

The study introduces a novel method to the thermal IRI, which allows for the elucidation of the functional link between several facial thermographic areas of interest. It has been discovered that the thermal seeds of the nose tip and the glabella are associated with face pixels in a precise way (butterfly-shaped). Correlating the GSR and its tonic component with all of the thermal signals of the facial IRI exhibited the same pattern. These findings proved the presence of a functional route that leads to the behavior described. The results were also supported by the findings of a pathological case study, whose correlation maps did not exhibit the same “butterfly” structure but rather had an asymmetrical pattern for each of the seed correlation maps. This is the first attempt of a functional diagnostic tool that may be utilized by neurologists, physiologists, and clinicians in general, in addition to being a novel and promising method to IRI processing.

## Data Availability

The data presented in this study are available on request from the corresponding author. The data are not publicly available due to privacy issues.

## Code availability

The codes developed for the purpose of the study are available on request to the corresponding author.
